# *Trichoderma gamsii* affected herbivore feeding behaviour on *Arabidopsis thaliana* by modifying the leaf metabolome and phytohormones

**DOI:** 10.1111/1751-7915.13310

**Published:** 2018-09-17

**Authors:** Dongmei Zhou, Xing‐Feng Huang, Jianhua Guo, Marcia L. dos‐Santos, Jorge M. Vivanco

**Affiliations:** ^1^ Department of Plant Pathology College of Plant Protection Nanjing Agricultural University Nanjing 210095 China; ^2^ Department of Horticulture and Landscape Architecture Center for Rhizosphere Biology Colorado State University Fort Collins CO 80523 USA; ^3^ Institute of Plant Protection Jiangsu Academy of Agricultural Sciences Nanjing China; ^4^ Department of Chemical and Biological Engineering Colorado State University Fort Collins CO 80523 USA; ^5^ Plant Molecular Biology Laboratory Department of Genetics – “Luiz de Queiroz” College of Agriculture – ESALQ University of Sao Paulo Piracicaba SP 13418‐900 Brazil

## Abstract

Plants can re‐programme their transcriptome, proteome and metabolome to deal with environmental and biotic stress. It has been shown that the rhizosphere microbiome has influence on the plant metabolome and on herbivore behaviour. In the present study, *Trichoderma gamsii* was isolated from *Arabidopsis thaliana* rhizosphere soil. The inoculation of roots of *Arabidopsis thaliana* with *T. gamsii* significantly inhibited the feeding behaviour of *Trichoplusia ni* and affected the metabolome as well as the content of phytohormones in *Arabidopsis* leaves. *T. gamsii*‐treated plant leaves had higher levels of amino acids and lower concentrations of sugars. In addition, *T. gamsii*‐treated plant leaves had more abscisic acid (ABA) and lower levels of salicylic acid (SA) and indole‐3‐acetic acid (IAA) in comparison with the untreated plants. Furthermore, the inoculation with *T. gamsii* on different signalling mutants showed that the induction of defences were SA‐dependent. These findings indicate that *T. gamsii* has potential as a new type of biocontrol agent to promote plant repellence to insect attacks.

## Introduction

The change of metabolite profile (Walling, 2000; War *et al*., 2012) is one of the strategies used by plants to defend against biotic and abiotic stresses. Many studies have indicated that plant‐beneficial microbe interactions can alter the plant metabolite profile and improve plant defence to insect attacks (van de Mortel *et al*., 2012; Estrada *et al*., 2013). *Trichoderma* species play an important role in suppression of plant disease by directly antagonizing with root and foliar pathogens as well as by inducing resistance in plants. The *Trichoderma*‐induced resistance in plants leads to physiological and biochemical changes including enhanced defence enzymatic activity, upregulation of defence‐related genes, and changes in metabolic profiles (Mastouri *et al*., 2010; Contreras‐Cornejo *et al*., 2011; Carreras‐Villasenor *et al*., 2012). A recent study showed that *Trichoderma harzianum* T22 can enhance the production of volatile organic compounds (VOCs) in tomato leading to an increased attractiveness towards aphid parasitoids (Coppola *et al*., 2017). A similar study showed that *T. atroviride* triggers maize defences against the insect herbivore *S. frugiperda* by the increased emission of volatile terpenes and accumulation of jasmonic acid (JA) (Contreras‐Cornejo *et al*., 2018).

Phytohormones, including JA, ethylene (ET), salicylic acid(SA), abscisic acid (ABA), auxin, cytokinins (CK), brassinosteroids (BR) and gibberllins (GB), have been reported to play important roles in both plant‐insect and plant‐microbe interactions (Erb *et al*., 2012; Lazebnik *et al*., 2014). These phytohormones function as signal transducers in plant‐induced defence pathways. For instance, JA and SA are the two major regulators of local and systemic defence responses (Pieterse *et al*., 2012; Soler *et al*., 2013). Other hormones, such as ET and ABA also modify the plant defence responses (van Loon *et al*., 2006; Ton *et al*., 2009; Robert‐Seilaniantz *et al*., 2011). Recently, it was found that application of the combination of JA and SA led to metabolic changes in the leaves and phloem of *Plantago lanceolata* and altered herbivory behaviour (Schweiger *et al*., 2014). Similar studies have shown that the phytohormone profiles of *Trichoderma*‐treated plants were modified leading to defence and growth promotion activity (Contreras‐Cornejo *et al*., 2011; Mathys *et al*., 2012; Martinez‐Medina *et al*., 2013). Co‐inoculation of *T. virens* and *T. atroviride* to *Arabidopsis* roots accumulated the canonical defence hormones SA and JA as well as camalexin, which conferred resistance against *Botrytis cinerea* (Contreras‐Cornejo *et al*., 2011).

*Trichoplusia ni* (Lepidoptera: Noctuidae), also known as the cabbage looper moth, has a broad range of host plants (Sutherland and Greene, 1984) and is a major and devastating pest in vegetable growing areas worldwide. To date, the control of *T. ni* excessively depends on the use of chemical insecticides which can damage the environment. In addition, the development of resistance to insecticides is increasing (Soares and Porto, 2007; Weddle *et al*., 2009). Application of biocontrol agents is an alternative to control this insect and other pests. Therefore, the purpose of this work was to characterize the components of a rhizosphere microbiome that was previously reported to have potential impact on the metabolome profile of *Arabidopsis* and negatively affected *T. ni* larvae feeding (Badri *et al*., 2013). In the present study, we isolated a fungus (*Trichoderma gamsii)* from *Arabidopsis thaliana* rhizosphere soil that altered the metabolome of *Arabidopsis* and negatively affected *Trichoplusia ni* larvae feeding when it was inoculated to the soil in close proximity to roots. We further studied the levels of phytohormones and plant metabolites in *Arabidopsis* to unravel the mechanisms behind the change of larvae feeding behaviour by *T. gamsii*.

## Results

### Fungal isolates altered herbivore feeding behaviour

**Figure 1:**
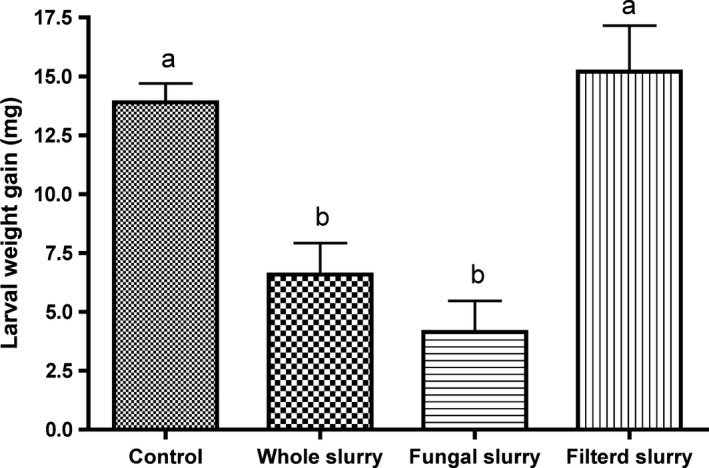
Larval gained weight of *Trichoplusia ni* fed for 24 h on four‐week‐old *Arabidopsis* plants amended with soil microbiomes. The control treatment received only Hoagland's solution. Each treatment had 18 pots (repetitions) and each pot contained one plant. The values represent the means ± SEM. Bars with dissimilar letters are significantly different (*P *< 0.05; Tukey's honest significance test).

A previous study showed that the microbiome from the *Arabidopsis* soil enhanced the plant resistance to *T. ni* herbivore (Badri *et al*., 2013). To determine if fungi and/or bacteria from the *Arabidopsis* soil played a role in changing the metabolome of *Arabidopsis* leaves, we treated the plants with whole slurry (containing all microbes), fungi‐enrichment‐slurry or filtered slurry (all microbes removed). We found the *T. ni* larval weights were significantly reduced when fed on plants treated with whole slurry and fungi‐enrichment‐slurry compared to those treated with filtered slurry and control plants (no treatment) (Fig. 1). Moreover, plants treated with whole slurry and fungi‐enrichment‐slurry did not have significant differences on gained larval weights. These findings indicate that fungi in the *Arabidopsis* soil played a key role in modulating the feeding behaviour of *T. ni*. Thus, we isolated fungal cultures from the soil slurry.

In total, 13 fungal isolates were obtained from the *Arabidopsis* soil and were screened by applying them individually to *Arabidopsis* roots to test the effects on the herbivore behaviour of *T. ni*. The relative gained weight of *T. ni* fed on *Arabidopsis* plants, inoculated with these fungal isolates individually, revealed that these fungal isolates have different effects on herbivore behaviour of *T. ni* (Fig. S1). Some isolates (e.g. F4‐2, F7, F14, and F16) showed positive effects on the gained weight of *T. ni*; while the isolate F18 had negative effects on the gained weight of *T. ni*. Meanwhile, the rest of the fungal isolates (e.g. F1, F3‐1, F10, F13, F15, F17 and F19) didn't have significant effects on the gained weight of *T.ni*. The fungal isolate F18 was selected for further experimentation because it consistently showed significant decrease of the larval weight gain of *T. ni* (Fig. S1).

**Figure 2:**
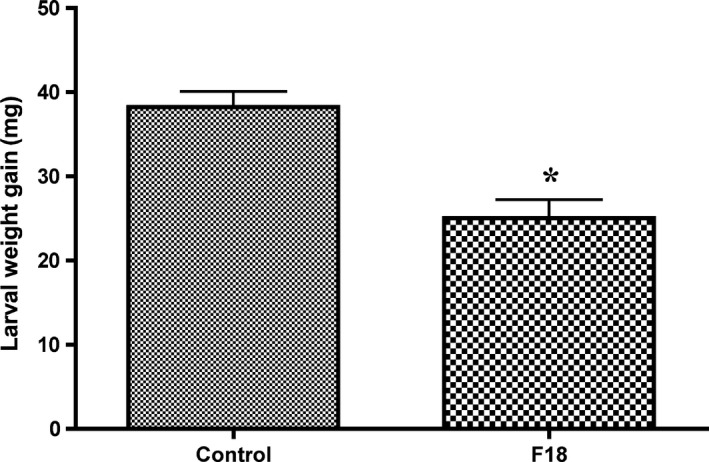
Larval gained weight of *Trichoplusia ni* fed for 24 h on four‐week‐old *Arabidopsis* Col‐0 plants amended with fungal isolate F18. The control treatment received only Hoagland's solution. Each treatment had 18 pots and each pot contained four plants. This experiment was repeated four times. All experiments showed the same trend. Data in this figure represent the results of one experiment. The values represent the means ± SEM. The asterisk above the bar indicates significance relative to the control at *p* < 0.05 level (*t*‐test).

We repeated the experiment four times to confirm the effects of F18 on the larval weight gain of *T. ni*. The results from these experiments showed the treatment with F18 had a significantly lower (40% decrease) larval weight gain compared to that of the control treatment (Fig. 2).

### Identification of the selected fungus by ITS sequencing

The highly conserved region of fungal rRNA gene of F18 was amplified and sequenced. The sequence was used in a BLAST (blastn) search using the NCBI nucleotide databases. The fungal isolate F18 was identified as *Tricoderma gamsii* with 100% sequence identity to *Trichoderma gamsii* strain DAOM 231637 (GenBank Accession: EU280129). Thus, we name the selected fungal isolate F18 as *Trichoderma gamsii* F18.

### Influence of *T. gamsii* F18 on the leaf metabolome

**Figure 3:**
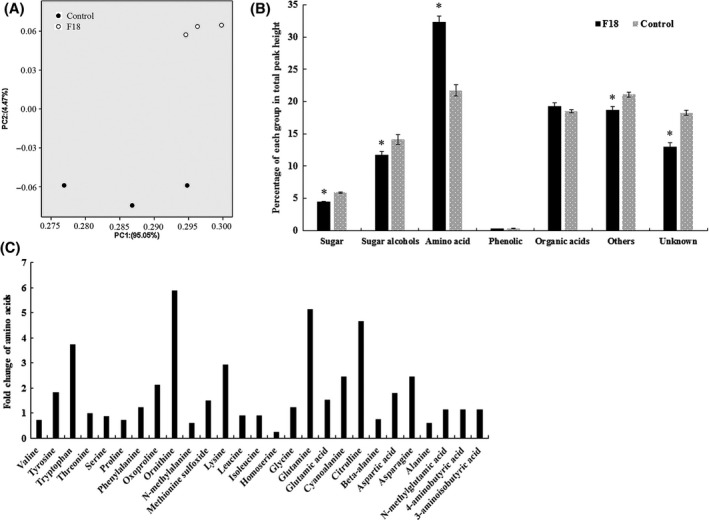
Leaf metabolites of *Arabidopsis* analysed by GC‐MS. A. Principal Component Analysis (PCA) of the leaf metabolites of *Arabidopsis* inoculated with and without *T. gamsii* F18. B. Metabolomics features detected by GC‐MS were categorized into six groups to generate a cumulative peak height for each group. The six groups included sugar, sugar alcohols, amino acid, phenolics, other compounds (fatty acid, amide and amine) and unknown (uncategorized compounds). The values represent the means SEM. The asterisks above the bars indicate significance relative to the control at *P* < 0.05 level (*t*‐test). C. Fold change of amino acids in *T. gamsii* F18‐treated leaves compared with control plants.

The influence of *T. gamsii* F18 on the leaf metabolites of *Arabidopsis* was analysed by GC‐MS. Based on GC‐MS analyses, 524 putative compounds were detected, and 177 compounds were annotated. Principle component analysis (PCA) showed that the metabolome of plants inoculated with *T. gamsii* F18 formed a distinct cluster in comparison with that of control plants (Fig. 3A). There were no significant differences in the contents of phenolic (*t*‐test, *P *=* *0.23) and organic acids (*t*‐test, *P *=* *0.27) between *T. gamsii* F18‐treated and untreated plants (Fig. 3B). However, the inoculation with *T. gamsii* F18 showed significantly lower levels of total sugars, sugar alcohols, other compounds including amide and amine, and unknown compounds (*t*‐test, *P *<* *0.05). On the contrary, the control plants exhibited less amino acids and their derivatives (*t*‐test, *P *<* *0.05) than plants inoculated with *T. gamsii* F18. Among the 27 detected amino acids, thirteen amino acids (tyrosine, tryptophan, serine, oxoproline, ornithine, phenylalanine, lysine, glutamine, glutamic acid, cyanoalanine, citrulline, aspartic acid and asparagine) increased their contents by more than 50% after *T. gamsii* F18 inoculation, while five amino acids (valine, N‐methylalanine, homoserine, beta‐alanine and alanine) were reduced by 25%–73% compared with the control plants (Fig. 3C).

### Influence of *T. gamsii* F18 on the content of plant phytohormones

Five phytohormones including indole‐3‐carboxylic acid (ICOOH), indole‐3‐acetic acid (IAA), jasmonic acid (JA), abscisic acid (ABA) and salicylic acid (SA) in *Arabidopsis* plants were detected and analysed by LC‐MS. As shown in Table 1, the inoculation with *T. gamsii* F18 resulted in different levels of phytohormones in *Arabidopsis* plants. For instance, the inoculation with *T. gamsii* F18 significantly increased the content of ABA (28% higher than that of control), but significantly decreased the content of IAA and SA (19% and 21% less than that of control, respectively). Furthermore, no significant effects on the contents of ICOOH and JA were found by the inoculation of *T. gamsii* F18.

**Table 1:** Phytohormones in *Arabidopsis* leaf tissue inoculated with *T. gamsii* F18 and control

	Indole‐3‐carboxylic acid (ng in extract)	Indole‐3‐acetic acid (ng in extract)	Jasmonic acid (ng in extract)	Abscisic acid (ng in extract)	Salicylic acid (ng in extract)
Control	33.78 ± 2.30	77.74 ± 5.03	26.91 ± 6.33	42.71 ± 1.72	629.01 ± 1.80
F18	36.10 ± 8.55	56.18 ± 1.70*	21.01 ± 2.92	50.77 ± 4.59*	498.16 ± 7.90*

The values represent the means ± SEM. The asterisks above numbers indicate significance relative to the control at the *P *<* *0.05 level (*t*‐test).

### Insect performance on signalling mutants

**Figure 4:**
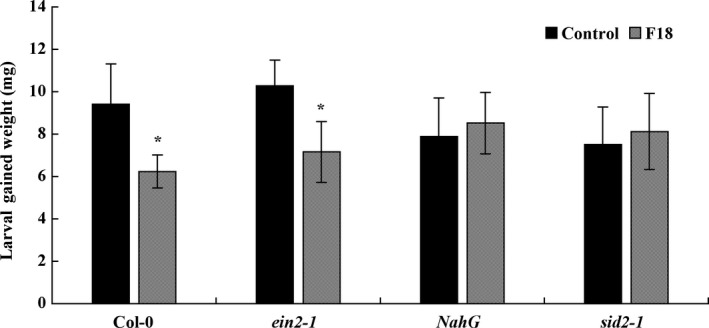
Larval gained weight of *Trichoplusia ni* fed for 24 h on four‐week‐old *Arabidopsis* Col‐0, *ein2‐1*,* NahG* and *sid2‐1* plants amended with *T. gamsii* F18 a week prior to insect herbivory. The control treatment received only Hoagland's solution. The values represent the means ± SEM. The asterisks above the bars indicate significance relative to the control at *P *< 0.05 level (*t*‐test).

We compared the difference of larval‐herbivore performance on *ein2‐1* (impaired in ET signalling pathway), *NahG* and *sid2‐1* (impaired in SA signalling pathway) plants after treatment with *T. gamsii* F18. The inoculation of *ein2‐1* with *T. gamsii* F18 resulted in 35% reduction in larval weight gain of *T. ni* compared to that of control (*ein2‐1* without *T. gamsii* F18 inoculation). In contrast, no significant difference in larval weight gain was found on *NahG* and *sid2‐1* plants inoculated with *T. gamsii* F18 compared to un‐inoculated controls (Fig. 4).

## Discussion

In the present study, we isolated 13 culturable fungal isolates from *Arabidopsis* soil and selected the fungus *T. gamsii* F18 which was found to modify the metabolome of *Arabidopsis* plants to make them less attractive to insects. Although other fungi present in the soil could have the potential to induce similar defence responses against *T. ni*, we only studied the culturable fungal isolates in this publication. Previous studies have shown that *Trichoderma* sp. have beneficial effects on plants. For instance, there are commercial agricultural products containing *T. atroviride, T. harzianum* and *T. hamatum* (Ha, 2010) that promote plant growth (Chang *et al*., 1986; Gravel *et al*., 2007; Contreras‐Cornejo *et al*., 2009), reduce the severity of plant diseases (Naseby *et al*., 2000; Nawrocka and Malolepsza, 2013) and enhance tolerance to abiotic stresses (Bae *et al*., 2009; Montero‐Barrientos *et al*., 2010; Dixit *et al*., 2011; Contreras‐Cornejo *et al*., 2014). In addition, *Trichoderma* sp. can control phytopathogenic nematodes such as *Rotylenchulus reniformis*,* Meloidogyne javanica* and *Meloidogyne cionopagum* (Sahebani and Hadavi, 2008; Bokhari, 2009). It also has been documented as a bio‐fungicide on flowers, ornamentals, vegetables, aromatic herbs and strawberries by the European Food Safety Authority (European Food Safety Authority, 2013). Our findings showed that the inoculation of *Arabidopsis* roots with *T. gamsii* F18 affected *T. ni* herbivory feeding behaviour on *Arabidopsis* leaves.

### *T. gamsii* F18 altered plant metabolome

Plant metabolites are modified by associated microbes such as endophytic fungi, beneficial soil‐borne microbes and plant growth promoting rhizobacteria (Pineda *et al*., 2010; van de Mortel *et al*., 2012; Estrada *et al*., 2013). In the *Trichoderma*‐plant interaction, it has been shown that the fungus re‐programmes the plant's transcriptome, proteome and metabolome leading to enhancement of the plant's ability to tolerate different types of stresses (Alfano *et al*., 2007; Shoresh *et al*., 2010).

In the present study, we observed that the *T. gamsii* F18‐treated leaves had less sugars than control plants. Sugars are essential for plant growth and development due to their roles as carbon and energy sources, signals and putative defence priming agents (Koch, 2004; Gomez‐Ariza *et al*., 2007). *T. gamsii* F18 may mediate the *T. ni* feeding behaviour by diminishing the attractiveness of the insect for primary metabolites such as sugars (Juniper and Richard, 1986). Another possibility is that *T. gamsii* F18 may be able to intensify the plant's immune reactions by reducing the sugar pools in favour of defence. Plant defence responses require the primary metabolites to support cellular energy and impose a fitness cost (Heil and Baldwin, 2002; Kangasjarvi *et al*., 2012).

Increasing evidence shows that amino acid signalling pathways may crosstalk with biotic and abiotic signalling networks in plants. Transcriptomics and metabolomics approaches used in *Arabidopsis* revealed that amino acids are differentially produced in *Arabidopsis* in response to pathogen and herbivory attack (Rojas *et al*., 2014). The biosynthesis of tryptophan, glutamine and glutamic acid are induced in herbivore‐infested plants. The overproduction of these amino acids seems to serve as precursors for inducible defence metabolites (Brotman *et al*., 2012; Rojas *et al*., 2014; Zhou *et al*., 2015). In our study, the leaves of *T. gamsii* F18‐treated plants had more tyrosine, tryptophan, serine, oxoproline, ornithine, phenylalanine, lysine, glutamine, glutamic acid, cyanoalanine, citrulline, aspartic acid and asparagine compared to the control plants. These amino acids are likely to account for plant defence in multiple manners.

In plant–herbivore interactions, amino acids function both as major growth‐limiting nutrients and as precursors for the generation of plant defence compounds. For example, tryptophan plays a critical role in regulating plant responses to biotic and abiotic stresses (Radwanski and Last, 1995; Zhao and Last, 1996; Sangha *et al*., 2011). Transcript profiling data generally show herbivory‐induced changes in the expression of genes related to the production of glutamine, glutamic acid, asparagine and aspartic acid (Thompson and Goggin, 2006; Caldana *et al*., 2011; Appel and Cocroft, 2014; Zhou *et al*., 2015), which are involved in nitrogen assimilation (Coruzzi and Last, 2000). Amino acids also play a role in signal transduction to induce defence gene expression. Glutamine plays a signalling role in the synthesis of other amino acids and regulates the expression of genes involved in plant immunity (Kan *et al*., 2015). Other amino acids induced in the leaves of *T. gamsii* F18‐treated plants, such as ornithine and oxoproline, are important intermediates in primary metabolism, and their roles in plant defence are unknown (Zeier, 2013). Decreased levels of valine, homoserine and alanine were observed in *T. gamsii* F18‐treated plants. There is limited information on the role of these amino acids on plant–insect interactions. Taken together, the induction of plant resistance to *T. ni* by *T. gamsii* F18 is related to the perturbation in particular amino acid homeostasis.

Phenolic compounds play important roles in plant defence responses during pathogen infection and herbivore attack because of their antioxidant properties (Kulbat, 2016). In our study, no significant difference was observed in total phenolics content between *T. gamsii* F18‐treated and untreated plants. However, it should be noted that SA and shikimic acid were dramatically lower in *T. gamsii* F18‐treated plants than in the control plants. The shikimic acid pathway provides important intermediates in terms of chorismic acid for the synthesis of aromatic acids (Tzin and Galili, 2010). The reduction of shikimic acid results in low levels of its derivatives. SA levels are indeed decreased in *T. gamsii* F18‐treated plants because its formation is through the side‐chain degradation of chorismic acid, which is an important intermediate of the shikimic acid pathway. SA is a key player in the regulation of plant‐induced resistance. Inoculation of *Trichoderma* has been observed to increase SA levels in cucumber plants (Segarra *et al*., 2007). However, SA levels were decreased by *T. gamsii* F18 in *Arabidopsis*; this might be due to a specific strain effect of *T. gamsii* F18.

Although there was no significant difference in organic acids levels between treatments, some were increased by *T. gamsii* F18 such as malic acid, citric acid and 2‐hydroxyglutaric acid. These acids are intermediate compounds in tricarboxylic acid cycle and are essential to generate cell energy to fuel metabolic reactions during plant defence responses (Agut *et al*., 2016; Groen, 2016).

### *T. gamsii* F18 affected phytohormones content in *Arabidopsis*

In addition to the metabolome alterations, *Trichoderma* isolates can also induce phytohormonal network changes on the host plant (Contreras‐Cornejo *et al*., 2011; Mathys *et al*., 2012; Martinez‐Medina *et al*., 2013, 2014). Studies have shown that the levels of defence‐ and stress‐related compounds such as JA, SA and ABA were enhanced in *Trichoderma*‐inoculated plants (Contreras‐Cornejo *et al*., 2011; Martinez‐Medina *et al*., 2011). Another study showed an increase in IAA induced by *Trichoderma* inoculation of Prunus and cherry rootstocks (Sofo *et al*., 2011). Consistent with previous studies, we also found that the application of *T. gamsii* F18 to the roots altered the content of IAA, SA and ABA in *Arabidopsis* leaves (Table 1).

IAA is the most common and important natural auxin in plants and plays an important role in plant development such as cell division, root initiation, emergence and shoot development (Phillips *et al*., 2011; Simon and Petrasek, 2011). However, unlike other reported *Trichoderma* strains that induced IAA levels in *Arabidopsis* and had growth promotion capacity (Contreras‐Cornejo *et al*., 2009; Martinez‐Medina *et al*., 2011), *T. gamsii* F18 did not increase the IAA content in our studies. This result might explain the fact that no plant growth promotion effect was observed with the inoculation of this strain (data not shown) indicating a different mode of action.

ABA is a major regulator of abiotic stress responses caused by drought, salt, cold and wounding (Christmann *et al*., 2006; Raghavendra *et al*., 2010), and also plays a prominent role in plant resistance to biotic stresses such as pathogen attack (Ton *et al*., 2009; Denance *et al*., 2013) and herbivorous insects (Bodenhausen and Reymond, 2007; Verhage *et al*., 2011; Pineda *et al*., 2012; Lazebnik *et al*., 2014). In our study, ABA content was increased in the *Arabidopsis* leaves when it was inoculated with *T. gamsii* F18. This result is consistent with previous studies that *Trichoderma* species increase ABA levels of host plants (Contreras‐Cornejo *et al*., 2011, 2015; Martinez‐Medina *et al*., 2011). Additionally, ABA has been shown to have positive regulatory effect on plants against insect feeding (Thaler and Bostock, 2004; Bodenhausen and Reymond, 2007). For example, Thaler and Bostock (2004) found that ABA‐deficient tomato plants were more susceptible to a chewing insect *Spodoptera exigua*. Bodenhausen and Reymond (2007) found that *Spodoptera littoralis* larvae performed better on *Arabidopsis* ABA‐biosynthetic mutant a*ba2‐1* and revealed a new role for ABA in defence against insects. In our case, the accumulation of ABA induced by *T. gamsii* F18 may be related to the negative regulation of the *T. ni* feeding behaviour.

JA and SA are dominant regulators of defence responses to herbivorous insects in plants (Glazebrook, 2005; Howe and Jander, 2008; Pieterse *et al*., 2009). In plant–insect interactions, the SA‐dependent pathway is induced by phloem‐feeding insects, whereas the JA‐dependent pathway is induced by chewing insects (Walling, 2000; De Vos *et al*., 2005; Glazebrook, 2005; Pieterse *et al*., 2012). Studies have reported that *Trichoderma* can activate both JA‐ and SA‐related signalling pathways in host plants (Segarra *et al*., 2007; Contreras‐Cornejo *et al*., 2011; Martinez‐Medina *et al*., 2011). The larval‐herbivore performance on SA mutants was not affected by *T. gamsii* F18 treatment further confirming that the resistance to *T. ni* attack induced by *T. gamsii* F18 is SA‐dependent.

Ethylene is a wound‐response regulator that is involved in plant defence against insects and also is involved in pathogen defence. For example, the *Arabidopsis ein2‐1* mutant had more damage than wild‐type plants upon *S. littoralis* attack (Stotz *et al*., 2000). In the present study, the inoculation of *ein2‐1* plants with *T. gamsii* F18 showed a significant decrease of larva weight gain compared with the untreated *ein2‐1* plants. This result suggests that *T. gamsii* F18 induces other defence pathways to affect *T. ni* feeding that are not related to ethylene. In this study, due to the reduced growth and plant size of ABA mutant *aba2‐1*, the seedlings were unable to be fed to *T. ni*. Thus, at this time, we cannot give an exact explanation on the role ABA related to the resistance induced by *T. gamsii* F18.

In conclusion, this study reports a newly isolated *Trichoderma gamsii* strain that could trigger changes on primary metabolites and alter the phytohormones especially SA and ABA in plant leaf tissues leading to enhanced resistance to insect attack. However, further studies are necessary to determine the effect of *T. gamsii* on multiple plant hosts. It should be noted that some PGPR have growth promotion effects on multiple plant species while others have effects on specific host plants (Herde and Howe, 2014).

## Experimental procedures

### Isolation and characterization of fungi

The microbiome was isolated from the *A. thaliana* soil reported in the study of Badri *et al*. (2013). Three different soil slurries including whole slurry (containing all microbes), fungi‐enrichment‐slurry or filtered slurry (all microbes removed) were applied to *A. thaliana* plants to determine what bio‐components were responsible for changing the metabolome of *Arabidopsis* leaves. The whole soil slurry was prepared according to the method described by Badri *et al*. (2013). The fungi‐enrichment‐slurry was prepared by incubating the whole slurry with an antibiotic cocktail containing four kinds of antibiotics (Rifampicin, 100 μg/ml; Kanamycin, 100 μg/ml; Erythromycin, 10 μg/ml; Spectinomycin, 100 μg/ml) for 48 h to eliminate the bacteria. The fungi‐enrichment‐slurry was applied to LB agar plates for 24 h to make sure there were no bacteria in the slurry. Then, the fungi‐enrichment‐slurry was filtered through Whatman Grade No. 1 filter paper to collect the fungal mycelia. The mycelia were washed with fresh Hoagland's solution twice to get rid of the antibiotic solution and re‐suspended in 500 ml Hoagland's solution. The filtered slurry was prepared by centrifuging the whole slurry at 12 000 rpm for 15 min and then passed through a 0.2 μm filter to remove all the microbes. The filtered soil slurry was plated on LB media to ascertain no microbial colonies were presented.

Surface‐sterilized *A. thaliana* (Col‐0) seeds were germinated on Murashige and Skoog medium (MS) (Murashige and Skoog, 1962) agar plates containing 1% sucrose for a week in a growth chamber (25°C, continuous light). Seven‐day‐old *Arabidopsis* seedlings were transferred into pots (6 cm × 3.6 cm × 6 cm) containing approximately 80 g of a mix of sterile sand and vermiculite (1:1 = v:v). Transferred seedlings grew in a growth chamber (25°C, 16 h/8 h light/dark, relative humidity of 80‐85%). Ten ml of the whole soil slurry, fungi‐enrichment‐slurry, the filtered slurry or the Hoagland's solution (as control) was inoculated to plants after 2 weeks of transplanting (three‐week‐old plants), respectively.

One week after inoculation, *Trichoplusia ni* larvae in the 5th‐instar stage were placed on the leaves of *Arabidopsis* plants. Each pot contained one plant and one larva. Larvae were weighed individually before feeding (initial weight) and after 1 day feeding (final weight). The gained weight of each larva was calculated by the final weight minus the initial weight. The experiment was conducted with 18 replicates per treatment.

### Isolation and screening of fungal isolates for inhibition of herbivore behaviour

The fungi‐enrichment‐slurry was prepared as the method described above. The fungi‐enrichment‐slurry was gradient‐diluted from 10^−1^ to 10^−4^ and streaked onto Potato Dextrose Agar (PDA) plates, and the plates were subsequently incubated for 5 days at 28°C. Single colonies of fungal isolates with different morphology were transferred to new PDA plates. Each fungal isolate was cultured on new PDA plates for at least three times to make sure it was a pure colony and stored at 4°C on PDA plates for use. In total, 13 fungal isolates were obtained.

The fungal isolates were cultured on PDA agar plates at 28°C for 5 days, and mycelia blocks were suspended in Hoagland's solution to get spore solution at a concentration of 1 × 10^6 ^CFU/ml. Three‐week‐old *A. thaliana* (Col‐0) seedlings were root‐inoculated with the fungal spores of the 13 isolates, respectively, suspended in 2 ml Hoagland's solution at a concentration 1 × 10^6 ^CFU/ml. The same amount of Hoagland's solution was applied to control plants. *T. ni* larvae in the 5th‐instar stage were transferred on the leaves of *Arabidopsis* plant after 1 week of fungal inoculation. Each pot contained one plant and one larva. Larvae were weighed individually before feeding (initial weight) and after 1 day feeding (final weight). The gained weight of each larva was calculated by the final weight minus the initial weight. The experiment was conducted with 18 replicates per treatment.

Based on the results of the screening study, fungal isolates which had significant effects on the herbivore behaviour of *T. ni* larvae were selected for further experimentation. To study the effect of those selected fungi on herbivore behaviour of *T. ni*, 2 ml spores at a concentration 1 × 10^6 ^CFU/ml of the selected fungi were applied to three‐week‐old *A. thaliana* (Col‐0) seedlings in pots (9.0 cm × 9.0 cm × 9.0 cm) using the experimental conditions described above. After a week of inoculation, larvae in the 5th‐instar stage were placed onto the leaves. There were nine pots for each treatment. Each pot contained four plants that were fed to one larva. The gained weight of each larva was calculated by the final weight minus the initial weight. This experiment was repeated four times.

### Identification of selected fungus

Total DNA of selected fungal isolates was extracted using a method described by Liu *et al*. (2000). The highly conserved fungal rRNA gene was PCR amplified using primers ITS1F (5′‐ TCCGTAGGTGAACCTGCGG‐3′) and ITS4 (5′‐ TCCTCCGCTTATTGATATGC‐3′) (White *et al*., 1990). The PCR products were sequenced at the Proteomics and Metabolomics Facility at Colorado State University. The ITS sequences were compared against the NCBI nucleotide databases using the standard nucleotide–nucleotide BLAST (blastn) search algorithm.

### Leaf metabolome

Twenty mg of freeze‐dried leaf tissue was finely grounded and extracted in 1 ml of 80% methanol. The cleaned extract was aliquoted into two equal portions, and the supernatant was dried down for further analysing. Internal standards C08‐C30 fatty acid methyl esters (FAMEs) were added, and the sample was derivatized by methoxyamine hydrochloride in pyridine and subsequently by N‐methyl‐N‐trimethylsilyltrifluoroacetamide for trimethylsilylation of acidic protons (Broeckling *et al*., 2005). Samples were analysed according to the methods of Feihn (Fiehn *et al*., 2008) by GC‐TOF primary metabolite analysis at West Coast Metabolomics Center, UC‐DAVIS. Mass spectrometry parameters were used as follows: a Leco Pegasus IV mass spectrometer was used with unit mass resolution at 17 spectra s^−1^ from 80–500 Da at −70 eV ionization energy and 1800 V detector voltage with a 230°C transfer line and a 250°C ion source. ChromaTOF versus 2.32 was used for data preprocessing. Apex masses were reported for using in the BinBase algorithm. Result files were exported to a data server with absolute spectra intensities and further processed by a filtering algorithm implemented in the metabolomics BinBase database. Quantification was reported as peak height using the unique ion as default, unless a different quantification ion is manually set in the BinBase administration software BinView. Raw data files were secured at the NIH Metabolomics database, http://www.metabolomicsworkbench.org.

### Phytohormone extraction

The phytohormone was extracted and analysed using the methods described by Huang *et al*. (2017). Briefly, 100 mg of finely ground plant tissue was transferred into a 1.5 ml tube. One mL cold extraction solvent (20:79:1, methanol: isopropanol: acetic acid, v:v:v) was added to the tube. The tubes were vortexed on medium speed for 120 min at 4°C. Then, the tubes were centrifuged at 4°C (13 000 g, 15 min), and the supernatants were transferred into a new 1.5 ml tube. The samples were dried under gentle nitrogen stream and dissolved in 100 μl solvent (50:50, acetonitrile: water with 0.1% formic acid). Tubes were stored at −80°C overnight and centrifuged at 4°C (13 000 g, 15 min) to remove precipitation. The supernatants were transferred into HPLC vial and analysed by LC‐MS/MS.

### LC‐MS/MS

Phytohormones were chromatographically separated using a Waters nanoAcquity UPLC system on a Waters Atlantis dC18 column (3 μM, 300 μM × 150 mm) held at 40°C. Samples were held at 4°C in the auto‐sampler. Water (A) and acetonitrile (B), both with 0.1% formic acid, were used as buffers. The flow rate was 11.5 μl/min, and injection volume was 1 μl. Each sample was injected twice and hormone levels averaged. Phytohormones were analysed by selected reaction monitoring (SRM) on a Waters Xevo TQ‐S mass spectrometer in both negative and positive ion modes. The UPLC gradient was as follows: time (*t*) = 0 min, 10% B; *t* = 0.5 min, 10% B; *t* = 5.5 min, 95% B; *t* = 7.5 min, 95% B; *t* = 8 min, 10% B. The column was equilibrated for three minutes before each injection.

### Insect performance on signalling mutants

Seeds of *ein2‐1* (an ethylene‐insensitive mutant) and *sid2‐1*(SA induction‐deficient mutant) were obtained from P. Reymond (University of Lausanne, Lausanne, Switzerland) (Bodenhausen and Reymond, 2007), and seeds of transgenic *NahG* lines (salicylic acid‐deficient *NahG* transgenic lines) (Delaney *et al*., 1994) were obtained from J. Vivanco's lab. Seven‐day‐old seedlings of all the mutants were transferred to pots (6 cm × 3.6 cm × 6 cm) containing sterile vermiculite and sand (1:1) and were grown in a growth chamber at the same conditions as above studies. Two ml of fungal mycelia solution (1 × 10^6 ^CFU/ml) was applied to each plant two times when they were 14‐day and 21‐day old, respectively. The larvae were randomly placed onto the leaves after 7 days of the second inoculation. There were nine pots for each treatment. Each pot contained four plants and one larva. The gained weight of each larva was calculated by the final weight minus the initial weight. This experiment was repeated two times.

### Statistical analysis

Tukey's HSD (Honestly Significant Difference) test and *t*‐test were conducted to compare the difference in larva weight gain of the treatments and controls (SPSS Student Version 16.0 18.). Statistical significant differences of identified leaf metabolites between treatment and control were determined by *t*‐test. Clustering of the leaf metabolites (based on the peak height) was performed by principal component analysis (PCA) using Vegan package in R (R Core Team, 2013).

## Conflict of interest

We have no declaration of conflict of interest.

## Supporting information


**Fig. S1.** Larval weight gain of *Trichoplusia ni* fed for 24 h on 4‐week‐old *Arabidopsis* Col‐0 plants amended with fungal isolates. The control treatment received only Hoagland's solution. Larval weight gain of *T. ni* feeding for 24 h on 4‐w‐old *Arabidopsis* Col‐0 plants amended with F18. A, first experiment; B, second experiment; C, third experiment. The values represent the means ± SDEM. The asterisk above the bar indicates significance relative to the control at *P *< 0.05 level (*t*‐test).

**Table S1.** List of compounds identified from *T. gamsii* F18‐ treated and un‐treated Arabidopsis leaves based on GC‐MS analyses.
