# Supplementary figures and images for: Trichoderma gamsii affected herbivore feeding behaviour on Arabidopsis thaliana by modifying the leaf metabolome and phytohormones
Source: Microb Biotechnol. 2018 Sep 17;11(6):1195–206. doi: 10.1111/1751-7915.13310 (PMC6196387; doi:10.1111/1751-7915.13310)

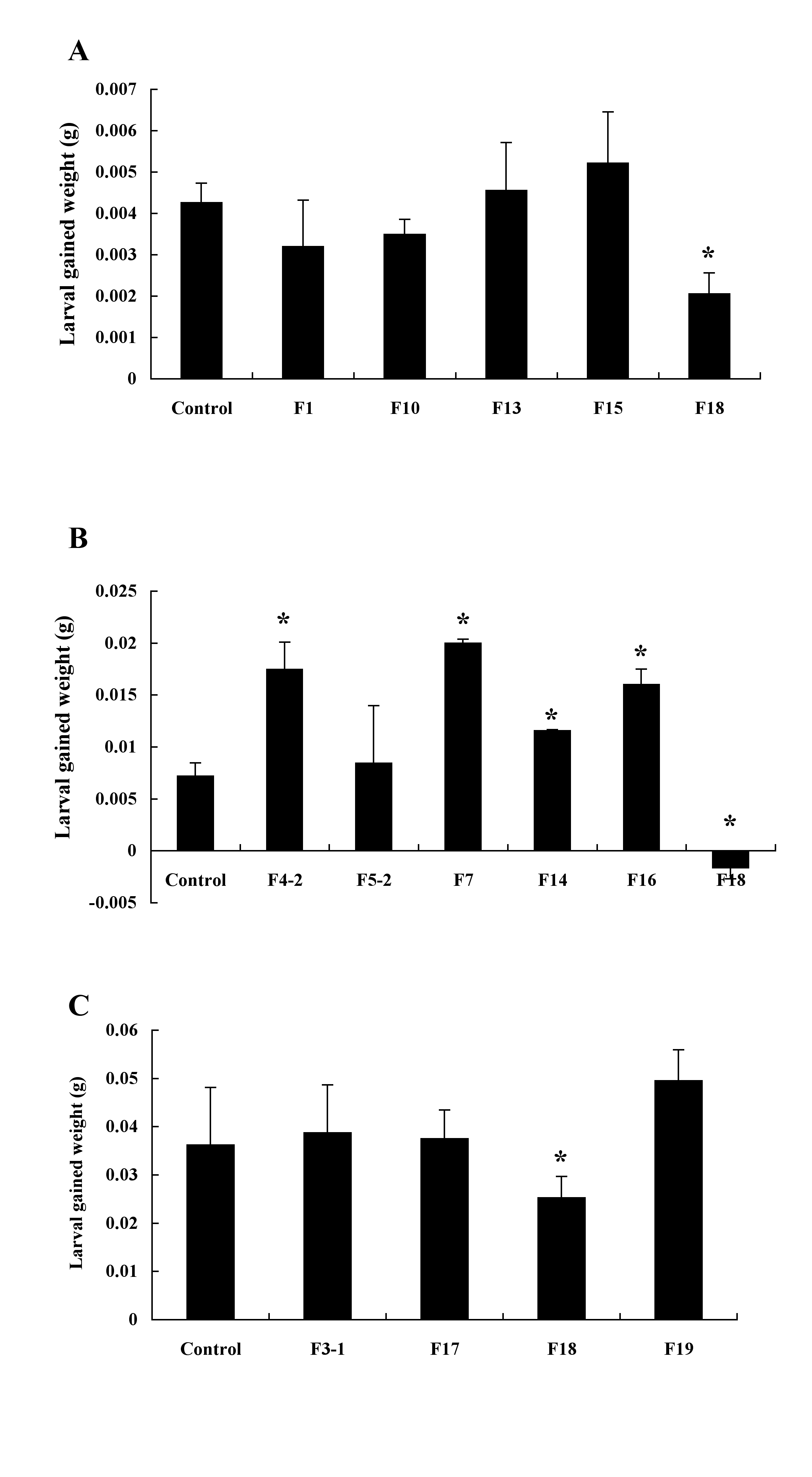

Supplement: Supplementary file 1 — Fig. S1. Larval weight gain of Trichoplusia ni fed for 24 h on 4‐week‐old Arabidopsis Col‐0 plants amended with fungal isolates. The control treatment received only Hoagland's solution. Larval weight gain of T. ni feeding for 24 h on 4‐w‐old Arabidopsis Col‐0 plants amended with F18. A, first experiment; B, second experiment; C, third experiment. The values represent the means ± SDEM. The asterisk above the bar indicates significance relative to the control at P < 0.05 level (t‐test). [file MBT2-11-1195-s001.tif]
